# IL‐17F induces IL‐6 via TAK1‐NFκB pathway in airway smooth muscle cells

**DOI:** 10.1002/iid3.149

**Published:** 2017-03-03

**Authors:** Masayuki Nakajima, Mio Kawaguchi, Kyoko Ota, Junichi Fujita, Satoshi Matsukura, Shau‐Ku Huang, Yuko Morishima, Yukio Ishii, Hiroaki Satoh, Tohru Sakamoto, Nobuyuki Hizawa

**Affiliations:** ^1^ Department of Pulmonary MedicineFaculty of MedicineUniversity of TsukubaIbarakiJapan; ^2^ Respiratory Disease CenterShowa University Northern Yokohama HospitalKanagawaJapan; ^3^ Asthma and Allergy CenterJohns Hopkins UniversityBaltimoreMarylandUSA; ^4^ National Health Research InstitutesTaipeiTaiwan

**Keywords:** IL‐17F, NF‐κB, TAK1

## Abstract

**Introduction:**

Interleukin (IL)‐17F plays a critical role in the pathophysiology of asthma. However, the precise role of IL‐17F in airway smooth muscle cells (ASMCs) and its regulatory mechanisms remain to be defined. Therefore, we sought to investigate the expression of IL‐6 by IL‐17F and the involvement of transforming growth factor β‐activated kinase 1 (TAK1) and nuclear factor (NF)‐κB by in ASMCs.

**Methods:**

ASMCs were cultured in the presence or absence of IL‐17F. The expression of IL‐6 gene and protein was analyzed using real‐time PCR and ELISA, and the activation of TAK1 and NF‐κB was detected by Western blotting. The effect of TAK1 inhibitor 5Z‐7‐oxozeaenol and NF‐κB inhibitor BAY 11‐7082 on the expression of IL‐6 was investigated. Finally, the short interfering RNAs (siRNAs) targeting TAK1 and a subunit of NF‐κB, p65 were transfected into ASMCs.

**Results:**

The expression of IL‐6 gene and protein was significantly induced by IL‐17F. IL‐17F activated TAK1 and NF‐κB in ASMCs. Transfection of siRNAs targeting TAK1 abolished IL‐17F‐induced phosphorylation of p65. Both 5Z‐7‐oxozeaenol and BAY 11‐7082 significantly inhibited IL‐17F‐induced IL‐6 production in a dose‐dependent manner. Similarly, transfection of the cells with siRNAs targeting TAK1 and p65 inhibited the expression of IL‐6.

**Conclusions:**

Collectively, these results provided evidence supporting the potential importance of the Th17‐ASMCs crosstalk via the IL‐17F‐IL‐6 axis in airway inflammation and as a candidate pharmacological target for airway inflammatory diseases such as asthma.

## Introduction

Asthma is characterized by the association of airway inflammation, airway obstruction, increased airway hyperreactivity, and remodeling. Airway smooth muscle is believed to contribute to the pathophysiology of asthma including direct causation of airflow obstruction via contraction. However, recent emerging evidences have demonstrated that airway smooth muscle plays a pivotal role in regulation of allergic airway inflammation via their ability to produce many inflammatory molecules including IL‐6 1, 2. IL‐6 is known to be a potential contributor of asthma pathogenesis. Intranasal administration of a blocking anti‐IL‐6 receptor antibody in a mouse model of asthma has been shown to be able to decrease eosinophilia, the expression of Th2 cytokines, and airway hyperresponsiveness 3. Hence, IL‐6 might be a suitable target for a new approach to asthma therapy. But, how ASMCs‐derived pro‐inflammatory mediators such as IL‐6 are regulated remain to be fully defined. Thus, further understanding of the mechanisms through which ASMCs‐derived IL‐6 is regulated would be important to uncover the pathogenic mechanisms of asthma.

Discovery of a novel molecule involving allergic airway inflammation and identification of its signaling mechanisms might help to clarify the pathogenesis of asthma. We and other groups cloned human IL‐17F gene 4, 5, 6. The IL‐17F gene is evidently upregulated in the airway from patients with asthma 4. Moreover, we have demonstrated that a single nucleotide polymorphism in IL‐17F gene that results in a loss‐of‐function mutation is inversely related to asthma risk and is a natural IL‐17F antagonist 7, 8. These findings indicate that IL‐17F is one of the important cytokines involved in the etiology of asthma. In addition, the level of IL‐17F expression is correlated with the disease severity of asthma 9. Although IL‐17F is able to act several cell types to induce various cytokines, the role of IL‐17F in ASMCs remains unclear 10, 11, 12. ASMCs express a receptor for IL‐17F, a heterodimer of IL‐17RA and IL‐17RC 13. However, its intercellular signaling pathway is not well defined. Recent studies have shown that transforming growth factor β‐activated kinase‐1 (TAK1), a member of the mitogen‐activated protein kinase kinase kinase family, is a pivotal signaling molecule leading to the activation of the transcription factors nuclear factor‐kappa B (NF‐κB) 14, 15. TAK1 regulates the pathogenesis of innate and adaptive immunity including airway inflammation. In bronchial epithelial cells, TAK1 mediates the signaling of environmental stimuli such as respiratory viral infection which is a major cause of acute exacerbations 16. However, the inducible factors of TAK1 in ASMCs remain unclear. In this study, we demonstrated, for the first time, that IL‐17F is able to activate the TAK1‐NF‐κB signaling pathway to induce IL‐6 expression in ASMCs.

## Methods

### Cell culture

ASMCs were purchased from Lonza (Walkersville, MD, USA) and cultured in SmBM medium with SmGM‐2 SingleQuots (Lonza, Tokyo, Japan) containing insulin, fibroblast growth factor, gentamicin, 5% fetal bovine serum, and epidermal growth factor at 37°C with 5% CO_2_ in humidified air. Confluent cells at passages 2–4 were used.

### Analysis of IL‐6 gene expression

Total RNA was extracted using RNeasy Mini Kit (Qiagen, Chatsworth, CA, USA) from 1 × 10^6^ ASMs at 4, 12, 24, and 48 h after stimulation with 10 and 100 ng/mL of IL‐17F (R&D Systems, Tokyo, Japan). cDNAs were synthesized from 1 µg of total RNA using the ReverTra Ace qPCR RT Kit (TOYOBO, Tokyo, Japan), followed by real‐time PCR. The sequences of primers for IL‐6 are as follows: forward, 5′‐AAAGAGGCACTGGCAGAAAA‐3′, reverse, 5′‐CACCAGGCAAGTCTCCTCAT‐3′; G3PDH: forward, 5′‐ACCACAGTCCATGCCATCAC‐3′, reverse, 5′‐TCCACCACCCTGTTGCTGTA‐3′. Real‐time PCR was done using a THUNDERBIRD SYBR qPCR Mix (TOYOBO), gene‐specific primers, and an ABI 7500 real‐time PCR system. The data were shown as fold induction relative to the control group. The values are expressed as mean ± SEM (n = 6 experiments).

### Analysis of IL‐6 protein production

Cell supernatants in ASMCs were harvested from cultures in the absence or presence of 10 and 100 ng/mL of IL‐17F at 4, 12, 24, and 48 h. Alternatively, the cells were also stimulated by 100 ng/mL of IL‐17A (R&D Systems) for 24 h. IL‐6 protein levels in the supernatants of IL‐17F‐stimulated cells were determined with a commercially available ELISA kit (R&D Systems) according to the manufacturer's instruction, and expressed as the amount recovered per 10^6^ cells. The values are expressed as mean ± SEM (n = 6 experiments).

### Detection of TAK1 and NF‐κB by Western blotting

For analysis of activation of TAK1 and NF‐κB, ASMCs were treated with IL‐17F (100 ng/mL) and in some cases with or without transfection with the siRNAs targeting TAK1, p65 and control using HiPerFect Transfection Reagent (Qiagen). The total cellular extracts (1 × 10^6^ cell equivalents/lane) were subjected to 7.5–15% Tris‐glycine gel electrophoresis (DRC, Tokyo, Japan), followed by transfer onto polyvinylidene difluoride membranes (Bio‐Rad, Tokyo, Japan). The Abs used were anti‐TAK1 Ab, anti‐phospho‐TAK1 Ab, anti‐phospho‐p65 Ab (Cell Signaling Technology, Danvers, MA, USA), anti‐p65 (Rel A) Ab (Santa Cruz Biotechnology, Santa Cruz, CA, USA).

### Effect of TAK1 inhibition on the expression of IL‐6

For analysis of the involvement of TAK1, ASMCs were treated in the presence or absence of a TAK1 inhibitor, 5Z‐7‐Oxozeaenol (Sigma–Aldrich, Tokyo, Japan) at varying doses, and a vehicle control, 0.1% DMSO, for 3 h before treatment with IL‐17F (100 ng/mL). The supernatants were harvested at 48 h after stimulation for analyses with ELISA. IL‐6 protein levels in the supernatants were determined as described above. These values are expressed as mean ± SEM (n = 6 experiments). Simultaneously, pre‐designed siRNAs for TAK1 (Santa Cruz Biotechnology) and control siRNAs (Ambion, Tokyo, Japan) were used. The siRNA transfection into ASMCs by HiPerFect Transfection Reagent (Qiagen) was performed according to the manufacturer's instruction. The supernatants were then harvested at 24 h after stimulation with 100 ng/mL of IL‐17F and subjected to analysis by ELISA, respectively. IL‐6 protein levels in the supernatants are expressed as mean ± SEM (n = 6 experiments).

### Effect of NF‐κB inhibition on the expression of IL‐6

For the analysis of involvement of NF‐κB, ASMCs were treated in the presence or absence of a NF‐κB inhibitor, BAY 11‐7082 (Calbiochem, Tokyo, Japan) at varying doses, and a vehicle control, 0.1% DMSO, for 1h before treatment with IL‐17F (100 ng/mL). The supernatants were harvested at 24 h after stimulation for analyses with ELISA. IL‐6 protein levels in the supernatants were determined as described above. These values are expressed as mean ± SEM (n = 6 experiments). Pre‐designed siRNAs for p65 (Santa Cruz Biotechnology) were also used for transfection into ASMCs as described above. The supernatants were then harvested at 24 h after stimulation with 100 ng/mL of IL‐17F and subjected to analysis by ELISA, respectively. IL‐6 protein levels in the supernatants are expressed as mean ± SEM (n = 6 experiments).

### Data analysis

The statistical significance of differences was determined by analysis of variance (ANOVA). The values are expressed as mean ± SEM from independent experiments. Any difference with *P*‐values less than 0.05 was considered significant. When ANOVA indicated a significant difference, the Scheffe *F*‐test was used to determine the difference between groups.

## Results

### Expression of IL‐6 gene and protein

**Figure 1:**
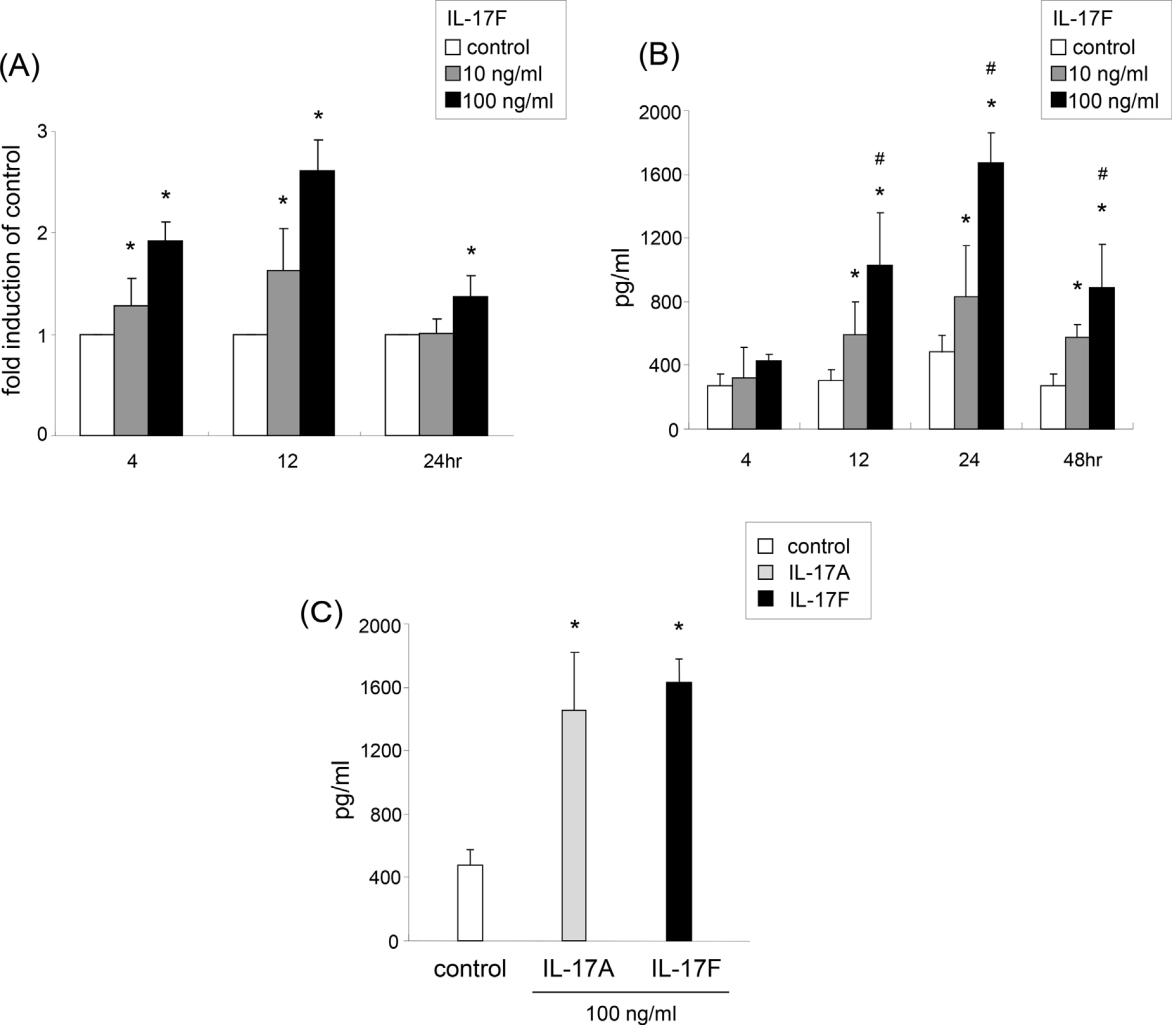
The expression of IL‐6 gene and protein by IL‐17F in ASMCs. (A) Gene expression of IL‐6 by IL‐17F. Real‐time PCR was performed as described in Materials and Methods. ASMCs were stimulated with IL‐17F (100 ng/mL) for 4, 12, and 24 h (n = 6). *P < 0.05 versus medium control. (B) IL‐6 protein expression by IL‐17F. ELISA was performed as described in Materials and Methods. The cells were stimulated with IL‐17F (100 ng/mL) for 4–48 h (n = 6). *P < 0.05 versus medium control. ^#^*P < 0.05 versus 10 ng/mL of IL‐17F‐stimulated cells. (C) IL‐6 protein levels induced by IL‐17A and IL‐17F in supernatants (n = 6). The cells were stimulated with 100 ng/mL of IL‐17A or IL‐17F for 24 h (n = 6). **P < 0.05 versus medium control.

A 100 ng/mL of IL‐17F significantly induced IL‐6 gene expression when compared with control at 4, 12, and 24 h time points (Fig. 1A). IL‐17F significantly induced its expression in a dose‐dependent manner at 4 and 12 h after stimulation. The levels of IL‐6 protein production were analyzed by ELISA (Fig. 1B). IL‐6 protein were detected in unstimulated cells, but its levels in supernatants were significantly increased, peaking at 24 h time point, in ASMCs stimulated with IL‐17F when compared with control at every time point. Similarly, IL‐17F significantly induced IL‐6 production in a dose‐dependent manner at 12, 24, and 48 h after stimulation. Other IL‐17 cytokine family, IL‐17A showed the similar potency to induce IL‐6 expression as IL‐17F at 24 h time point (Fig. 1C).

### Activation of TAK1 by IL‐17F

**Figure 2:**
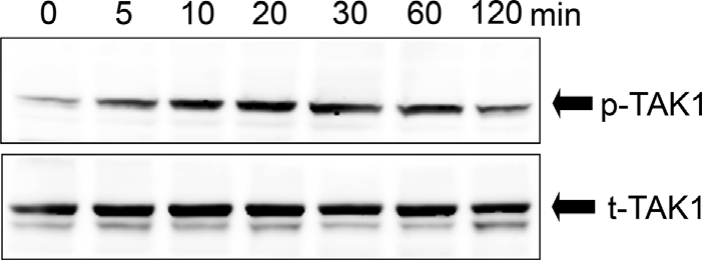
Activation of TAK1 by IL‐17F in ASMCs. The cells were incubated with IL‐17F (100 ng/mL) for different time points as indicated. Western blotting analysis was performed with Abs against total (t)‐TAK1 and phosphorylated (p)‐TAK1 as indicated. These results shown are representative of three separate experiments.

TAK1 was equally detected at all‐time points (Fig. 2). In contrast, a transient phosphorylation of TAK1 was seen on stimulation of the cells with IL‐17F, reaching the maximum at 10–20 min after stimulation and returned to baseline levels by 120 min in ASMCs.

### Effect of TAK1 inhibition on IL‐17F‐induced IL‐6 expression

**Figure 3:**
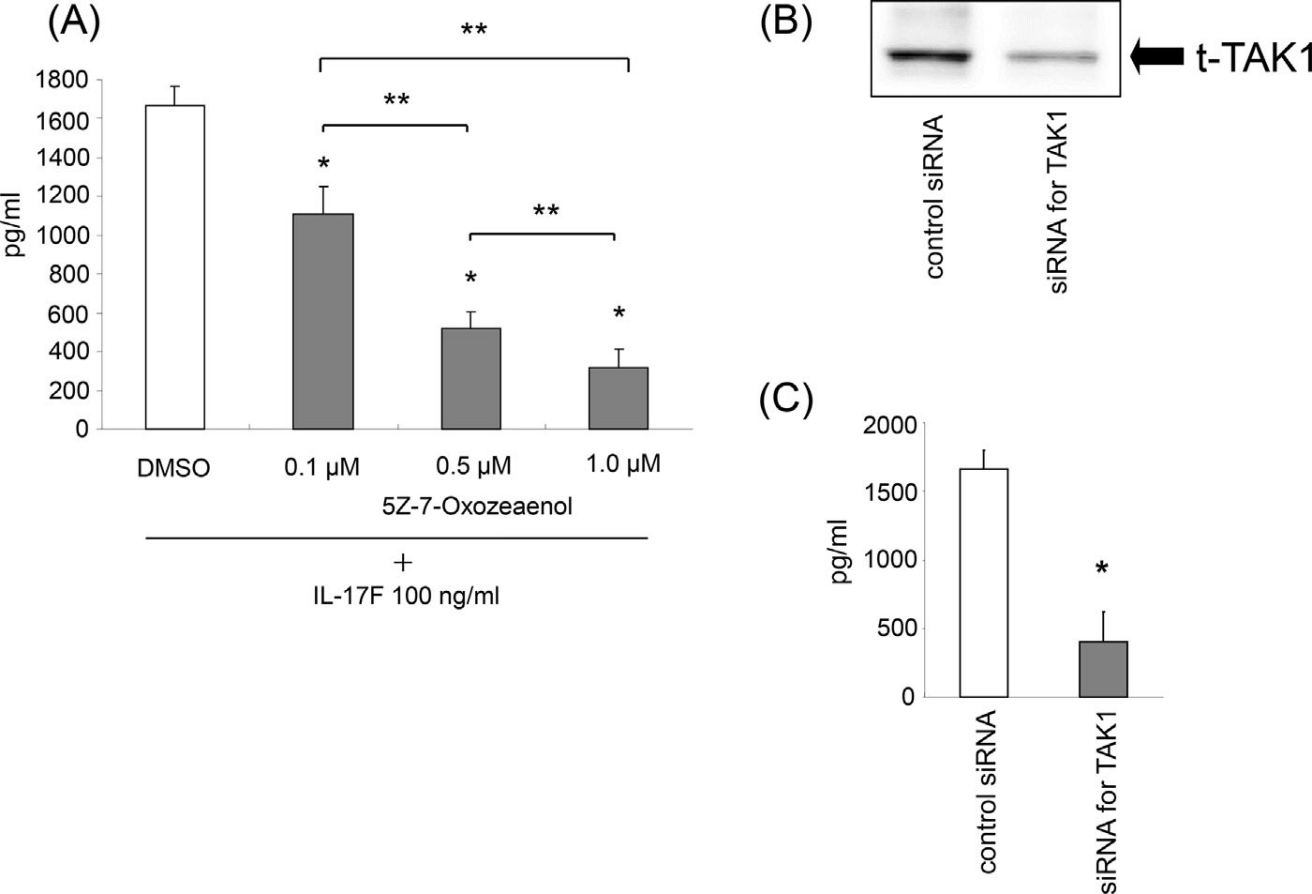
Effect of the inhibition for TAK1 on IL‐6 expression. ASMCs were pretreated with 5Z‐7‐Oxozeaenol (0.1, 0.5, and 1.0 µM) for 3 h before the 24 h‐stimulation of IL‐17F (100 ng/mL), and then IL‐6 protein levels in supernatants were measured by ELISA. The values are expressed as means ± SEM (n = 6). *P < 0.05 versus IL‐17F‐stimulated cells in the absence of the inhibitor. *P < 0.05 versus the presence of individual inhibitor. (B) The validation of blocking by siRNAs targeting TAK1 was performed by Western blotting. These results shown are representative of three separate experiments. (C) After transfection of the siRNAs, ASMCs were stimulated with IL‐17F (100 ng/mL) for 24 h. The levels of IL‐6 protein production in the supernatants were measured by ELISA. The values are expressed as means ± SEM (n = 6). *P < 0.05 versus IL‐17F‐stimulated cells transfected with a control siRNA.

**Figure 4:**
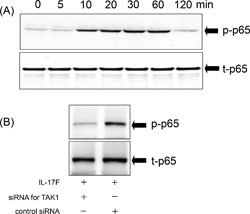
Activation of NF‐κB by IL‐17F. (A) Kinetic activation of NF‐κB by IL‐17F in ASMCs. The cells were incubated with IL‐17F (100 ng/mL) for different time points as indicated. Western blotting was performed with Abs against total (t)‐p65 and phosphorylated (p)‐p65. (B) Effect of siRNAs targeting TAK1 on IL‐17F‐induced phosphorylation of p65. The cells were transfected with siRNAs targeting TAK1 or control siRNAs, and then ASMCs were stimulated with IL‐17F for 30 min. Western blotting analysis was performed with Abs against t‐p65 and p‐p65. The results shown are representative of three separate experiments.

Pretreatment of the cells with 0.1, 0.5, and 1.0 µM of TAK1 inhibitor, 5Z‐7‐Oxozeaenol significantly blocked IL‐17F‐induced IL‐6 production in a dose‐dependent manner, while 3‐h pretreatment of the cells with vehicle alone (DMSO) did not affect its production in ASMCs (Fig. 3A). To further confirm whether TAK1 plays a role in IL‐17F‐induced IL‐6 expression, total TAK1 expression was diminished in the cells by transfecting with siRNAs targeting TAK1 (Fig. 3B). As shown in Figure 4C, IL‐6 production induced by IL‐17F was significantly inhibited in cells transfected with siRNAs targeting TAK1 when compared with cells transfected with a control siRNAs.

### Activation of NF‐κB (p65) by IL‐17F

A subunit of NF‐κB, p65, was detected at all‐time points (Fig. 4A). Phosphorylation of p65 was evidently detected at 10–60 min, and returned to baseline levels by 120 min in IL‐17F‐stimulated ASMCs (Fig. 4A). To establish the interrelationship between TAK1 and NF‐κB, the cells were transfected with siRNAs targeting TAK1 before the stimulation with IL‐17F. Transfection with the siRNAs clearly diminished the activation of p65 induced by IL‐17F, while transfection of control siRNAs did not affect the activation of p65 (Fig. 4B).

### Effect of NF‐κB inhibition on IL‐17F‐induced IL‐6 expression

**Figure 5:**
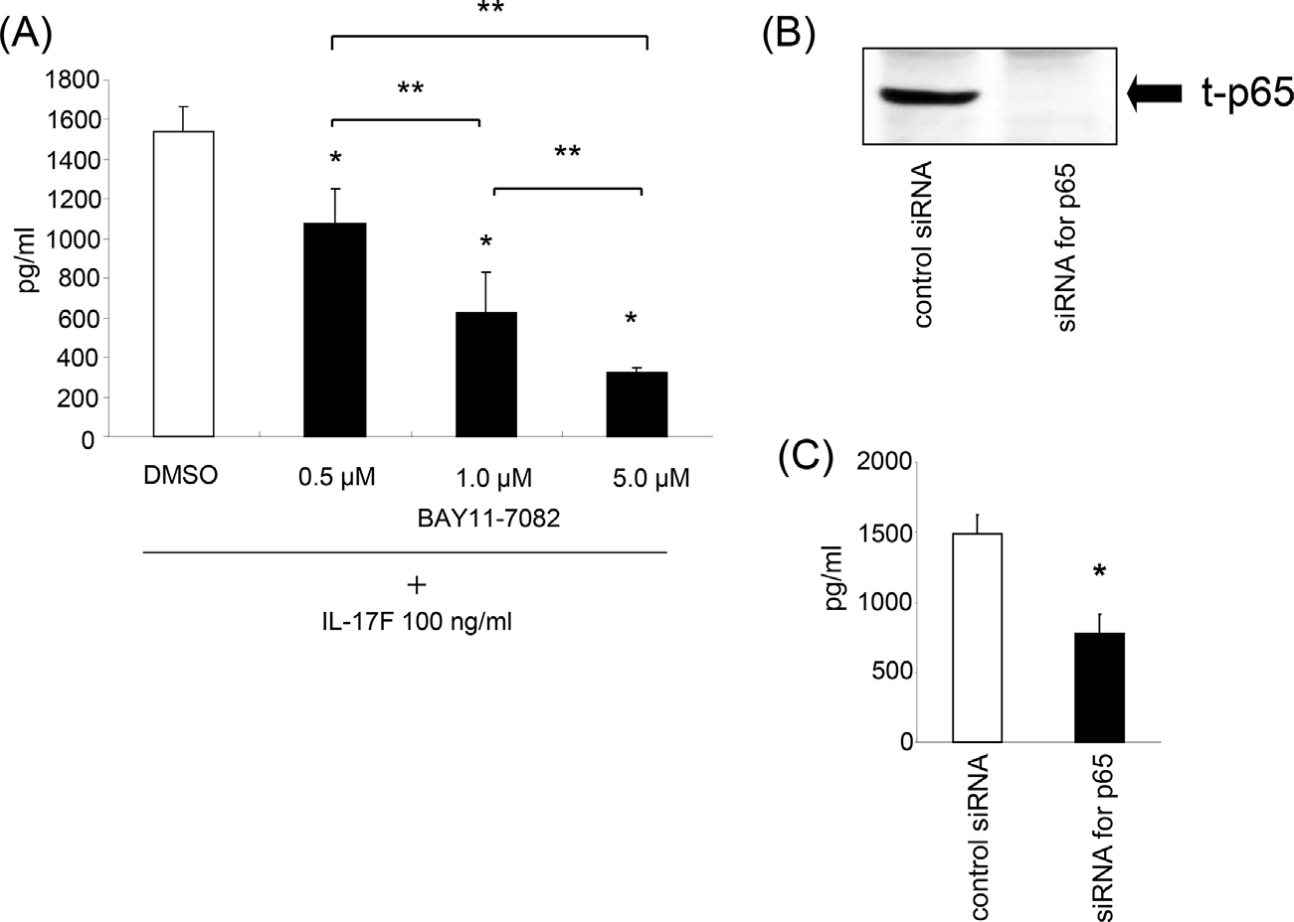
Effect of NF‐κB inhibition on IL‐17F‐induced IL‐6 expression. (A) ASMCs were pretreated with NF‐κB inhibitor, BAY 11‐7082 for 1 h before the 24 h‐stimulation of IL‐17F (100 ng/mL), and then IL‐6 protein levels in supernatants were measured by ELISA. The values are expressed as means ± SEM (n = 6). *P < 0.05 versus IL‐17F‐stimulated cells in the absence of the inhibitor. **P < 0.05 versus the presence of individual inhibitor. (B) The validation of blocking by siRNAs targeting p65 was performed by Western blotting. These results shown are representative of three separate experiments. (C) After transfection of the siRNAs, ASMCs were stimulated with IL‐17F (100 ng/mL for 24 h. The levels of IL‐6 protein production in the supernatants were measured by ELISA. The values are expressed as means ± SEM (n = 6). *P < 0.05 versus IL‐17F‐stimulated cells transfected with control siRNAs.

Pretreatment of ASMCs for 1 h with 0.5, 1.0 and 5.0 µM of a NF‐κB inhibitor, BAY 11‐7082 significantly decreased the levels of IL‐17F‐induced IL‐6 protein production in a dose‐dependent manner, while 1 h pretreatment of the cells with vehicle alone (DMSO) did not affect IL‐6 production (Fig. 5A). Finally, to further confirm whether NF‐κB plays a role in IL‐17F‐induced IL‐6 expression, it was found that when NF‐κB p65 was knocked down in the cells with siRNAs (Fig. 5B), IL‐6 production induced by IL‐17F was significantly inhibited when compared with cells transfected with control siRNAs (Fig. 5C).

## Discussion

In this study, we demonstrated that IL‐17F significantly induced the expression of IL‐6 in AMSCs via the activation of TAK1‐NF‐κB signaling pathway. These findings indicate the likely importance of the IL‐17F‐IL‐6 axis in airway inflammation as a consequence of the Th17‐ASMC crosstalk.

Current study demonstrated that IL‐17F signal mediates TAK1‐NF‐κB pathway. This suggests that this signaling pathway is pivotal for IL‐17F‐induced IL‐6 expression in ASMCs. TAK1 is for the first time, identified as a novel signaling molecule involved in the function of IL‐17F. Consistent with the previous studies, TAK1 is located upstream of NF‐κB, since a TAK1 inhibitor 5Z‐7‐Oxozeaenol diminished the phosphorylation of NF‐κB 15. Moreover, the activation of NF‐κB is crucial for IL‐6 expression by IL‐17F, since NF‐κB inhibitor and the specific siRNAs abrogated its expression. Recent reports demonstrated that TAK1 is clearly involved in the pathogenesis of airway inflammation. TAK1 activation in bronchial epithelial cells was induced by several inflammatory stimuli such as RS virus, *Pseudomonas aeruginosa* and diesel exhaust particles that are able to exacerbate airway inflammation 16, 17, 18. Interestingly, TAK1 is involved in steroid responsiveness in asthma 19. Blocking of TAK1 recovers cellular response to steroids in the presence of pathogenic bacteria via the regulation of MAPK phosphatase‐1activation. However, the functional role of TAK1 in ASMCs still remains. It is known that TAK1 contributes to cigarette smoke‐induced IL‐8 production and the causing of airway remodeling through the induction of growth factor‐induced proliferation of ASMCs 20, 21. Here, we reported that IL‐17F induced phosphorylation of TAK1, indicating that TAK1 is a potentially pharmacological target for the IL‐17F‐mediated airway inflammatory diseases. However, the inhibitors and siRNAs targeting TAK1 or NFκB‐p65 did not completely inhibit IL‐17F‐induced IL‐6 expression in this study. This suggests that the involvement of other signaling molecules. Further study is needed to identify novel signaling pathways of IL‐17F and its functional impact on IL‐6 expression.

IL‐17F shows several distinctive features of asthma such as airway remodeling, goblet cell hyperplasia, and increasing airway hyperreactivity. Overexpression of IL‐17F following Ag challenge in the airway of mice resulted in the induction of goblet cell hyperplasia and, gene expression of MUC5AC and significant increase in airway hyperreactivity 22. These findings suggest that IL‐17F can provide an additive effect on antigen‐induced allergic inflammatory responses. In addition, IL‐17F is associated with airway neutrophilia. Although neutrophilic inflammation is one of the features of severe asthma, its precise mechanism has not been clarified. Overexpression of IL‐17F in the mouse airways results in an increased number of neutrophils in bronchoalveolar lavage fluid (BALF) 22, 23. Interestingly, IL‐17F is more involved in airway neutrophilic inflammation to *Aspergillus oryzae* when compared with IL‐17A 24. Moreover, IL‐17F is able to induce CXC chemokines, such as IL‐8, ENA‐78, and GROα, that are potent chemoattractants for neutrophils 4, 25. Hence, Neutrophil recruitment into the airway may be regulated via, at least partially, IL‐17F‐induced CXC chemokines. IL‐17F‐producing cells are known to come from many cell types, such as bronchial epithelial cells, basophils, mast cells, γδT cells, CD8^+^T cells, and Th17 cells 4, 26, 27, 28. Especially, Th17 cells are deeply involved in the pathogenesis of a diverse group of immune‐mediated diseases, including asthma 29. Th17 cells are the major cell source of IL‐17F, and our current study demonstrated that IL‐17F could induce IL‐6 expression. IL‐6 is necessary for Th17 development through the induction of a transcriptional factor, retinoic acid receptor‐related orphan nuclear receptor (RORγt) 30. Although the cell source of IL‐6 in airway inflammation has not been fully understood, the current study suggests that IL‐6 is derived from, at least partially, ASMCs in response to IL‐17F. Taken together, it is possible that as a mode of the Th17‐ASMC crosstalk, IL‐17F‐induced IL‐6 further promotes Th17 cell differentiation, which, in turn, establishes a positive feedback loop resulting in the amplification of the Th17 response. Further in vivo study is needed to clarify the importance of the IL‐17F/IL‐6 axis in asthma.

The role of ASMCs in the pathogenesis of airway inflammation has become increasingly clear. ASMCs are able to induce a wide range of cytokines and chemokines that orchestrate airway inflammation 31. However, the functional role of IL‐17F in ASMCs has not been clarified. A few studies have demonstrated that IL‐17F induces the migration and proliferation of ASMCs 13, 32. These findings suggest the involvement of IL‐17F in airway remodeling. Interestingly, unlike IL‐17A, IL‐17F did not affect ASM cell contraction 33. However, the reason of this difference has not been dissolved yet. Although both IL‐17A and IL‐17F bind to same receptor the heterodimeric complex of IL‐17RA and IL‐17RC, the binding affinity of IL‐17A and IL‐17F is quite different 34. IL‐17RA effectively binds to IL‐17A. In contrast, it binds to IL‐17F with extremely lower affinity. The relative binding affinity of IL‐17F to IL‐17RC is more potent than to IL‐17RA. These findings may affect their different biological activity for ASM cell contraction. Further study is needed in the future.

On the other hand, IL‐6 is a key factor in the pathophysiologic events of asthma 35. Increased levels of IL‐6 are observed in serum, BALF, and induced sputum from asthmatic patients. The levels of IL‐6 in sputum and serum are inversely correlated with lung function in asthmatic patients 36, 37. Besides inducing Th17 cell development, IL‐6 is a critical regulator of promoting IL‐4 production during Th2 differentiation, inhibiting Th1 differentiation, and is a co‐factor in IL‐4‐dependent IgE synthesis 38, 39. Moreover, IL‐6 is involved in airway mucus hypersecretion that is one of the features of asthma 40. Hence, ASMCs are important target cells for IL‐17F, and contribute to the pathogenesis of allergic airway inflammation, at least partially, via the induction of IL‐6 expression.

In conclusion, we demonstrated that IL‐17F activates TAK1‐NF‐κB signaling pathway to induce IL‐6 expression. IL‐17F/IL‐6 axis might be involved in the pathophysiology of allergic airway inflammation, and targeting IL‐17F and its signaling pathways could be a novel strategy for asthma.

## Conflict of Interest

None declared.
